# Revisional Gastric Bypass Is Inferior to Primary Gastric Bypass in Terms of Short- and Long-term Outcomes—Systematic Review and Meta-Analysis

**DOI:** 10.1007/s11695-018-3300-2

**Published:** 2018-05-11

**Authors:** Michał Pędziwiatr, Piotr Małczak, Mateusz Wierdak, Mateusz Rubinkiewicz, Magdalena Pisarska, Piotr Major, Michał Wysocki, W.Konrad Karcz, Andrzej Budzyński

**Affiliations:** 1 2nd Department of General Surgery, Jagiellonian University Medical College, Krakow, Poland; 2 Centre for Research, Training and Innovation in Surgery (CERTAIN Surgery), Krakow, Poland; 3 Department of General-, Abdominal-, Vascular-, Thoracic- and Transplantation Surgery, Ludwig-Maximilians-University of Munich, Munich, Germany

**Keywords:** RYGB, Bariatric surgery, Revisional surgery, Obesity, Gastric bypass

## Abstract

**Purpose:**

Although Roux-en-Y gastric bypass (RYGB) is the main primary bariatric procedure, it has also been utilized as revisional bariatric surgery. Our aim is to compare revisionary gastric bypass with primary gastric bypass through systematic review with meta-analysis.

**Methods:**

Available literature was searched for eligible studies up to December 2017. Inclusion criteria were reports on morbidity, %EWL, or diabetes remission. Secondary outcomes involved mortality, anastomotic leakage, operative time, and length of hospital stay. Random effect meta-analyses were undertaken.

**Results:**

Initial search yielded 1164 references. Final meta-analysis involved 21 studies and revealed significant differences in terms of morbidity (RR1.54, *p* < 0.001) and EWL (WMD-19.9, *p* < 0.001). There were no differences in diabetes remission.

**Conclusion:**

Revisionary RYGB has worse weight loss effect with greater morbidity rate than primary RYGB.

**Electronic supplementary material:**

The online version of this article (10.1007/s11695-018-3300-2) contains supplementary material, which is available to authorized users.

## Introduction

A significant proportion of patients undergoing bariatric procedures fail to achieve their weight loss goal, regain weight, or develop procedure-related complications. The failure rate is estimated to be as high as 40% and is closely related to primary procedure, patients’ characteristics, and their compliance with postoperative dietary habits [1–3]. According to published data, up to a quarter of patients undergoing bariatric surgery will require revisional surgery within 10 years after primary treatment [4, 5]. Given the increasing number of primary bariatric procedures performed worldwide, it may lead to a significant clinical problem.

For a long time, the Roux-en-Y gastric bypass (RYGB) has been the gold standard in the treatment of obesity and remains one of the most commonly performed bariatric procedures. Its excellent weight loss effect, together with successful impact on obesity-related comorbidities, has been well documented in the literature [6]. The RYGB has also been utilized as revisional surgery after failed primary procedure, with encouraging results. However, there are still no clear guidelines on the optimal revisional procedure. This is mostly due to limited data on long-term results of revisional procedures. Moreover, revisional surgeries are more complex [7, 8]. Only recently have long-term outcomes of revisional and primary RYGB been compared. To evaluate safety and results, we have attempted a systematic review of the available literature in order to assess the morbidity, mortality, and long-term results of revisional RYGB in comparison to that of primary RYGB.

## Methods

### Study Selection

A systematic review of the literature was performed using the Medline, Embase, and Cochrane databases to identify all eligible studies that compared patients undergoing primary RYGB with revisionary Roux-en-Y gastric by-pass (RRYGB). The used search terms included the following: “revision,” “reoperation,” “re-do,” “gastric by-pass,” “LRYGB,” “RYGB,” “primary,” “original,” and “first.” These terms were combined using Boolean operators “AND” and “OR.” Some references of the acquired articles were also located manually. The most recent search was performed on 12 December 2017. Ovid search strategy is available in supplementary file 1.

Studies eligible for further analysis had to fulfill the following criteria: (1) comparison of EWL between patients undergoing RYGB and RRYGB or (2) an objective evaluation of overall morbidity or (3) diabetes mellitus (DM) remission (4) publication in English. Studies were excluded when there was (1) lack of comparative data, (2) lack of primary outcomes or insufficient data to analyze, and (3) a procedure other than RYGB.

### Outcomes of Interest

Primary outcomes of interest were overall morbidity, % of lost excess weight (EWL), and DM remission. Secondary outcomes of interest involved mortality rate, anastomotic leakage rate, hypertension remission, operative time, and length of hospital stay.

### Data Extraction and Quality Assessment

All references were reviewed and evaluated by two teams of two researchers. In case of any doubts about eligibility for inclusion, an attempt was made to reach consensus within the group. If no resolution was possible, an arbitrary decision was made by another reviewer. Data from included studies were extracted independently by all teams. Only full-length articles were eligible for extraction. When available, the following data were extracted: first author, year of publication, country, number of operated subjects, and outcomes of interest.

Non-randomized studies were evaluated according to the Newcastle–Ottawa Scale (NOS), which consists of three factors: patient selections, comparability of study groups, and assessment of outcomes. A score of 0 to 9 was assigned to each study, and studies achieving a score of 6 or higher were considered high-quality. This study was performed according to the Preferred Reporting Items for Systematic Reviews and Meta-Analyses (PRISMA) guidelines and Meta-Analysis of Observational Studies in Epidemiology (MOOSE) consensus statement [9, 10]. The study was registered in the PROSPERO Database and the assigned number is CRD42018087537.

### Data Analysis

Analysis was performed using RevMan 5.3 (freeware from The Cochrane Collaboration). Statistical heterogeneity and inconsistency were measured using Cochran’s Q tests and I2, respectively. Qualitative outcomes from individual studies were analyzed to assess individual and pooled risk ratios (RR) with pertinent 95% confidence intervals (CI) favoring patients undergoing revisionary surgery, and by means of the Mantel–Haenszel random-effects method. When appropriate, mean and standard deviation were calculated from medians and interquartile ranges using a method proposed by Hozo et al. [11]. Weighted mean differences (WMD) with a 95%CI are presented for quantitative variables using the inverse variance random-effects method. Statistical significance was observed with two-tailed 0.05 level for hypotheses and with 0.10 for heterogeneity testing, while unadjusted *p* values were reported accordingly. Dichotomous outcome analysis involved subgroup analysis for case-control and case-matched studies. EWL analysis involved subgroups in regard to the length of follow-up, 1 and 2 years.

## Results

**Fig. 1:**
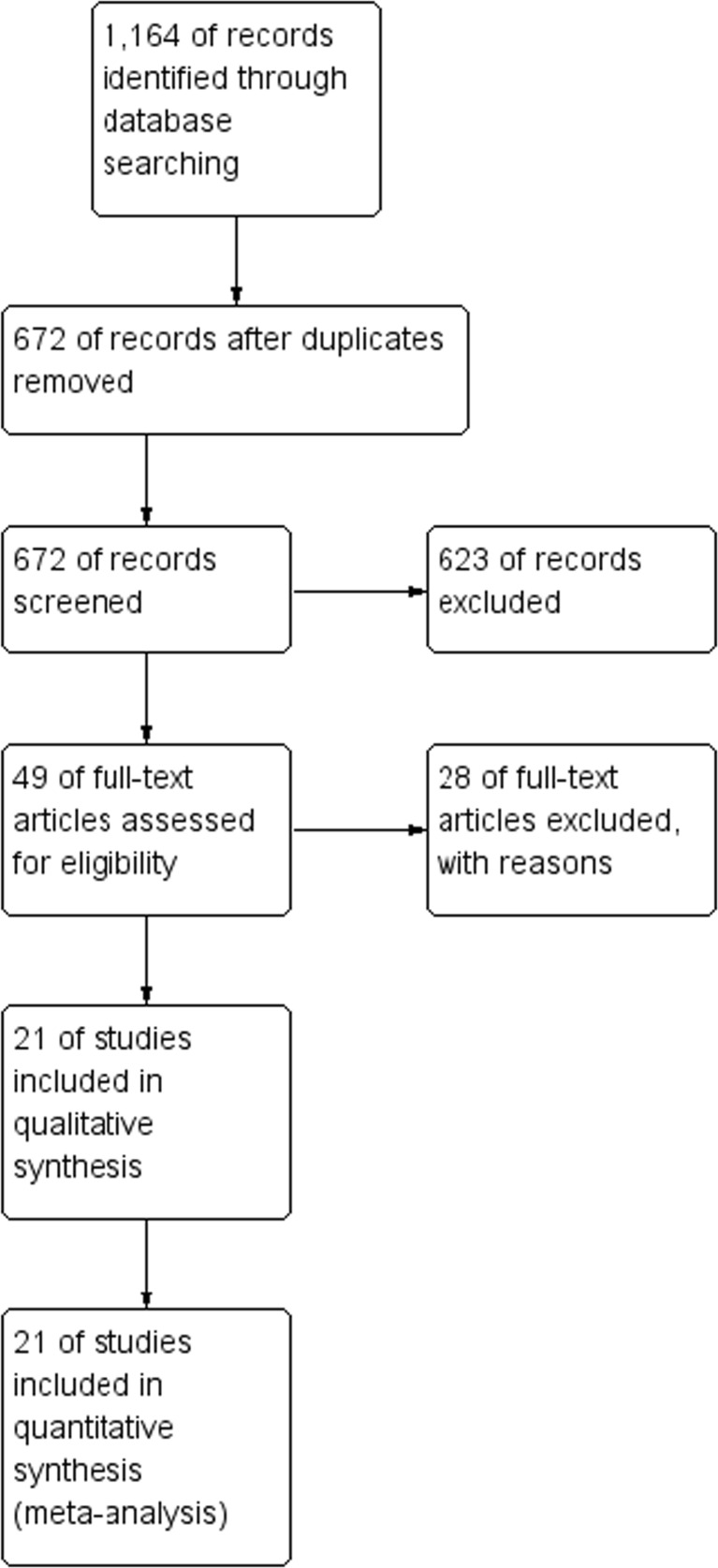
PRISMA flowchart

An initial reference search yielded 1164 articles. After removing 492 duplicates, 672 articles were evaluated through titles and abstracts. This produced 49 papers suitable for full-text review. Finally, we narrowed this down to 21 studies eligible for data extraction, with a combined total of 14,763 patients (3043 in RRYGB group and 11,720 in RYGB group) [1, 12–31]. A flowchart of the analyzed studies is presented in Fig. 1. Quality of the analyzed studies is moderate, with majority scoring at least 7 points according to NOS. Baseline information about the analyzed studies is presented in Table 1. The funnel plot of publication bias is presented in supplementary file 2. The cone is symmetrical which suggests low risk of publication bias. BMI prior to surgery was reported in 18 studies (45.3 vs. 43.3 kg/m^2^). Baseline BMI in patients undergoing revisional surgery (before primary procedure) was reported only in 8 of 21 analyzed studies, and also, no significant differences were observed (48.3 vs. 46 kg/m^2^, *p* = 0.14).

**Table 1:** Baseline information

Study	Year	Type	Country	Access	Total number of patients	Primary surgery	Quality score according to NOS scale
Topart [29]	2008	CC	France	Lap	259	AGB	7
Cadière [14]	2010	CC	Belgium	Lap	470	AGB, VBG	7
Radtka [23]	2010	CC	USA	Lap/open	928	VBG, RYGB	8
Zingg [31]	2010	CM	Australia	Lap/open	122	AGB, VBG, RYGB, SG	9
Deylgat [19]	2012	CC	Belgium	Lap/open	724	AGB, VBG, SG, RYGB, BPD-DS	8
Slegtenhorst [26]	2012	CC	The Netherlands	Lap/open	292	AGB	8
Stefanidis [1]	2013	CC	USA	ND	1206	AGB	7
Mor [22]	2013	CM	USA	ND	111	MGB, VBG, AGB, RYGB, SG, JIB	8
Thereaux [28]	2014	CC	France	Lap	1008	AGB	7
Thereaux [27]	2014	CM	France	Lap	90	AGB	6
Zhang [30]	2014	CM	USA	Lap/open	344	RYGB, VBG, AGB, SG	7
Delko [18]	2014	CM	Switzerland	Lap	96	AGB	9
Mohos [21]	2014	CM	Hungary	Lap	88	AGB, SG, RYGB, VBG	9
Sadot [25]	2015	CM	Israel	Lap	126	AGB	7
Coblijn, de Raaff [16]	2016	CC	The Netherlands	Lap	1130	ABG, SG	8
Coblijn, Lagarde [17]	2016	CC	The Netherlands	Lap	1667	SG, ABG	7
Raftopoulos [24]	2016	CC	Greece	Lap	820	ND	4
Al-Kurd [12]	2017	CM	Israel	Lap	322	AGB	7
Axer [13]	2017	CM	Sweden	Lap/open	4836	VBG, AGB, GB, SG, GBP, JIB	8
Chowbey [15]	2017	CM	India	Lap	60	SG, AGB	7
Malinka [20]	2017	CM	Switzerland	Lap	64	SG	7

*CC* case control, *CM* case matched, *AGB* laparoscopic adjustable gastric banding, *VBG* vertical banded gastroplasty surgery, *RYGB* Roux-en-Y gastric bypass, *SG* sleeve gastrectomy, *BPD-DS* biliopancreatic diversion with duodenal switch, *MGB* mini-gastric bypass, *JIB* jejunal–jejunal bypass, *GB* fixed gastric banding, *ND* no data

**Fig. 2:**
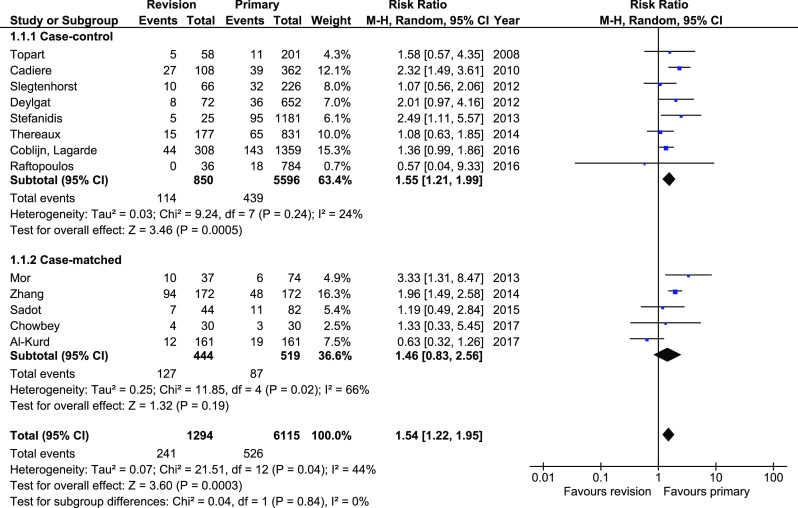
Pooled estimates of morbidity rate comparing revisionary gastric bypass versus primary gastric bypass. CI confidence interval, df degrees of freedom

Morbidity was reported in 13 studies, of which 5 were case-matched. Analysis revealed higher rate of complications in revisionary patients (241/1294, 18.6% in RRYGB vs. 526/6115, 8.6% in RYGB). In total, there were statistically significant differences between analyzed groups (RR 1.54, 95%CI 1.22–1.95, *p* = 0.0003); however, subgroup analysis did not find any differences in case-matched studies (95%CI 0.83–2.56, *p* = 0.19). The overall heterogeneity was moderate, *I*^2^ = 44% (Fig. 2).

**Fig. 3:**
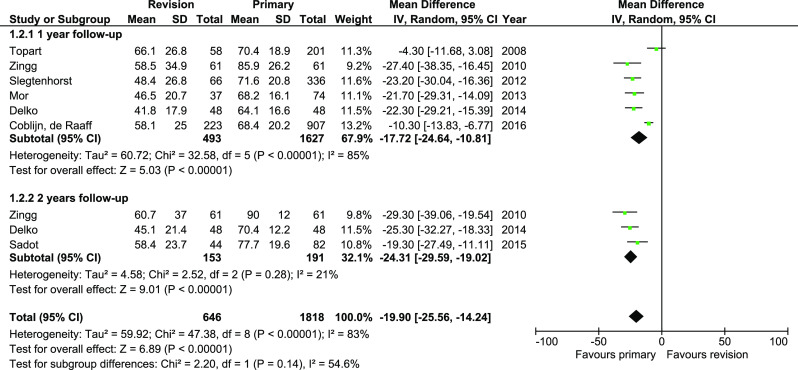
Pooled estimates of %EWL comparing revisionary gastric bypass versus primary gastric bypass. CI confidence interval, df degrees of freedom

Weight loss was reported in 15 studies; however, some of them reported different periods of follow-up. To avoid potential bias caused by this, for meta-analysis, we chose publications which reported 1- and 2-year periods of follow-up. In the end, we included seven studies. Analysis revealed significant differences in weight loss between groups (WMD − 19.9, 95%CI − 25.56–− 14.24). Subgroup analysis showed similar results. The heterogeneity in total and in 1-year group was very high. Sensitivity analysis did not find study generating inconsistence (Fig. 3).

**Fig. 4:**
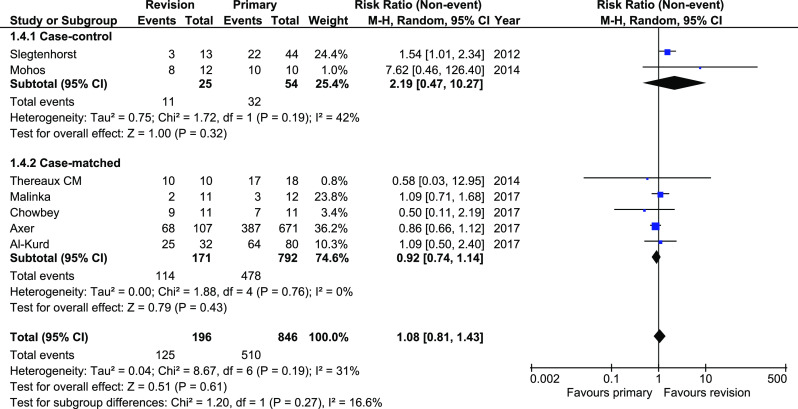
Pooled estimates of diabetes mellitus remission comparing revisionary gastric bypass versus primary gastric bypass. CI confidence interval, df degrees of freedom

DM remission was reported in seven studies. There were no significant differences in analyzed material, both in total and in subgroups (RR 1.05, 95%CI 0.81–1.43, *p* = 0.61). The heterogeneity in case-control subgroup was moderate, whereas in case-matched subgroup, there was no heterogeneity (Fig. 4).

**Fig. 5:**
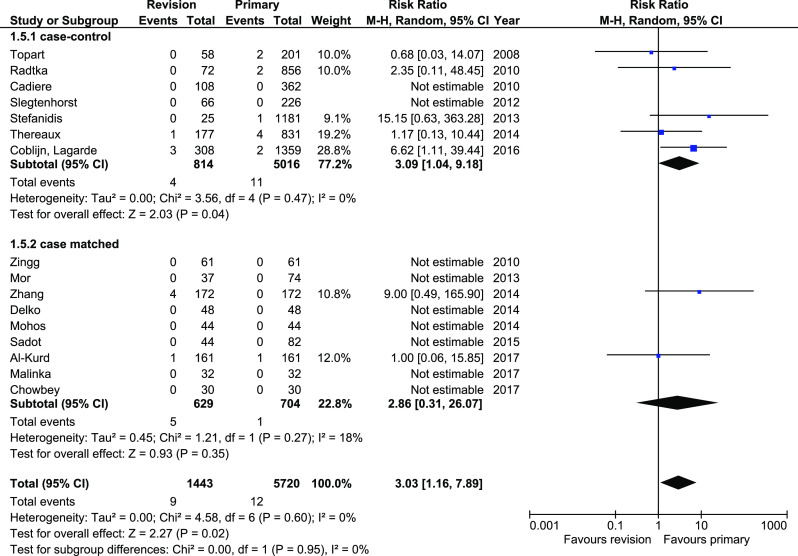
Pooled estimates of mortality rate comparing revisionary gastric bypass versus primary gastric bypass. CI confidence interval, df degrees of freedom

Mortality was reported in 16 studies. Mortality rate was significantly greater in revisionary group, 9/1443 (0.62%) versus 12/5720 (0.21%); RR 3.03, 95%CI 1.16–7.89, *p* = 0.02. However, subgroup analysis revealed no differences in case-matched subgroup (95%CI 0.31–26.07), whereas the low end of 95%CI was just slightly above 1 (95%CI 1.04–9.18). The heterogeneity in this outcome was low, both within total and within subgroups (Fig. 5).

**Fig. 6:**
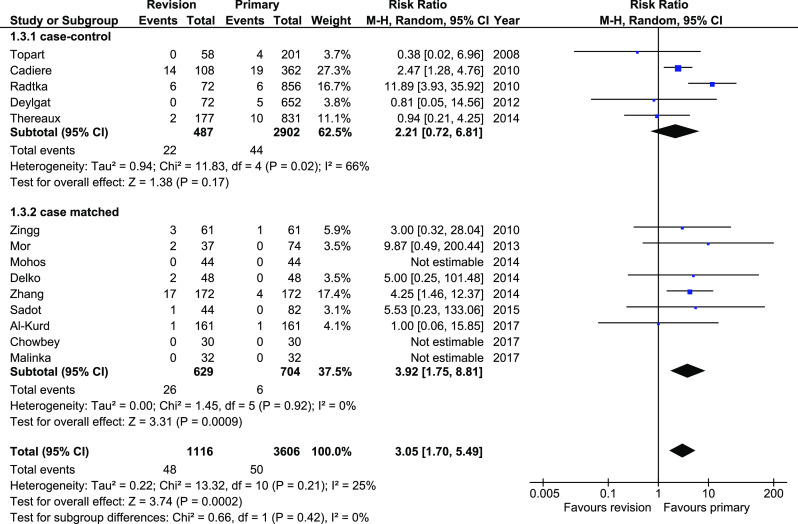
Pooled estimates of anastomotic leakage comparing revisionary gastric bypass versus primary gastric bypass. CI confidence interval, df degrees of freedom

Anastomotic leakage was reported in 14 studies. Analysis revealed significant differences in total (RR 3.05, 95%CI 1.7–5.49, *p* = 0.0002) and in case-matched subgroup (RR 3.92, 95%CI 1.75–8.81, *p* = 0.0009). The heterogeneity in total and in case-matched subgroup was low (Fig. 6). There were no significant differences within case-control subgroup (95%CI 0.72–6.81).

**Fig. 7:**
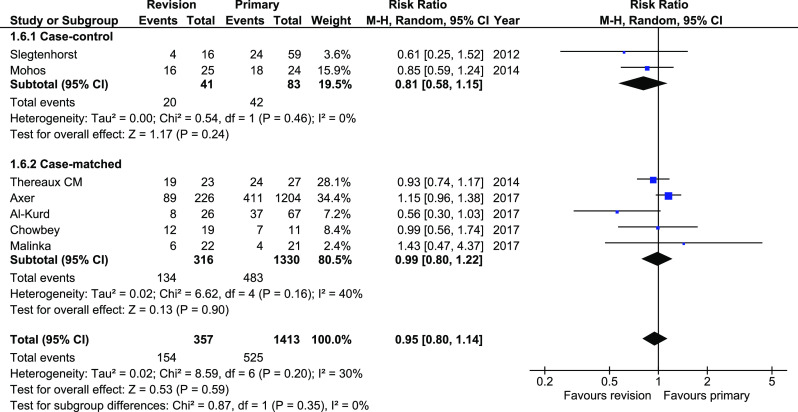
Pooled estimates of hypertension remission rate comparing revisionary gastric bypass versus primary gastric bypass. CI confidence interval, df degrees of freedom

Hypertension remission was reported in seven studies. There were no significant differences between analyzed group, both within total and within subgroups, 154/357 (43.14%) versus 525/1413 (37.15%), 95%CI 0.8–1.14 (Fig. 7). The heterogeneity was moderately low in case-matched subgroup and low in case-control subgroup.

**Fig. 8:**
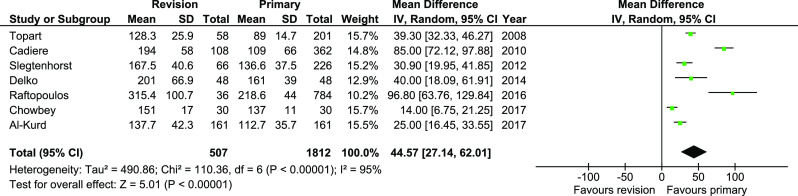
Pooled estimates of operative time comparing revisionary gastric bypass versus primary gastric bypass. CI confidence interval, df degrees of freedom

Operative time was reported in seven studies. Primary surgeries were significantly shorter by 44.57 min (WMD 44.57, 95%CI 27.14–62.01, *p* = 0.00001). However, the heterogeneity was very high and sensitivity analysis did not provide resolution (Fig. 8).

**Fig. 9:**
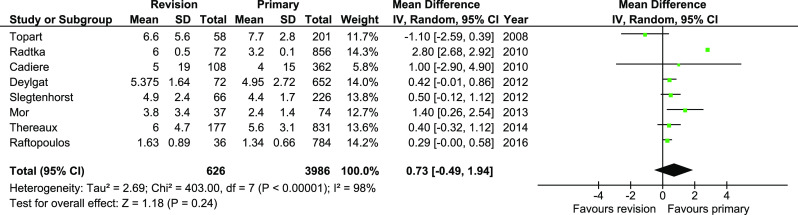
Pooled estimates of length of hospital stay comparing revisionary gastric bypass versus primary gastric bypass. CI confidence interval, df degrees of freedom

Length of hospital stay was reported in eight studies. There were no significant differences (95%CI − 0.49—1.94, *p* = 0.24); however, the heterogeneity was very high, *I*^2^ = 98% (Fig. 9).

## Discussion

To our knowledge, this is the first systematic review comparing outcomes of primary and revisional laparoscopic Roux-en-Y gastric bypass (LRYGB). The main findings show that revisions are associated with higher overall morbidity, increased mortality, and worse weight loss effect when compared to primary procedures. Operations last longer, but they are not associated with longer hospital stay. In addition, there were no differences in the postoperative effect on the resolution of obesity-related complications, such as diabetes mellitus or hypertension.

The majority of patients in the investigated studies underwent revision after adjustable gastric band (ABG) or vertical banded gastroplasty (VBG). It is in line with previous observations that showed disappointing results and failure rate up to 60% in long-term observation. This has led to a rapid decline in the number of ABG performed in recent years [32, 33]. Nevertheless, the number of ABG patients remains high, and one can expect that the majority of them at some point will require revision. We observed that morbidity, including anastomotic leakage, is higher in patients undergoing revisional LRYGB. This is in line with the study by Worni et al., who used the Nationwide Inpatient Sample (not included in our review due to exclusion criteria) and observed an increased number of adverse events (OR 8.0) [34]. In our review, the difference was lower (RR 1.54); however, it is still present. Interestingly, when a subgroup of case-matched patients was analyzed, no difference in morbidity was noted. In the majority of the studies, adverse events were reported up to 30 days after surgery, which may have introduced bias. The reported leakage rate of 1.39% after primary and 4.3% after revisions is within the range reported by other investigators [35–38].

Moreover, we noticed an increased mortality rate in the revisional group, but as in the case of morbidity, this increase was not present when case-matched studies were analyzed separately. On the one hand, this unequivocally confirms the higher risk of revisional surgery; on the other—the numbers are still low (0.2 vs. 0.6% mortality). Wide 95% confidence intervals (1.16–7.89) confirm the fragility of this finding. Our results show that every revisional patient should be well informed about the potentially higher risk of adverse events during the second procedure.

The main purpose of this review is to answer the question whether LRYGB may serve as a revisional procedure. Although weight loss was found in the majority of studies, meta-analysis including all of them was not possible due to differences in follow-up intervals and differences in reporting weight loss (simple weight loss, excess weight loss, excess body mass index loss, etc.). For these reasons, we were only able to group studies that reported EWL after 12 and 24 months. Patients after RRYGB lost on average 20% less of the excess weight than after primary procedure. It is an important observation taking into consideration that the preoperative BMI of the patients was comparable (45.3 vs. 43.3 kg/m^2^). However, baseline BMI in patients undergoing revisional surgery (before primary procedure) was reported only in 8 of 21 analyzed studies. There were also no significant differences (48.3 vs. 46 kg/m^2^, *p* = 0.14). Therefore, these results cannot be taken universally. It is likely that patients after revision had higher initial BMI, which might have influenced long-term outcomes because preoperative BMI is a well-known variable that is associated with postoperative weight loss [39].

Not all studies reported resolution of obesity-related diseases. Among those that did, we were not able to find any differences in meta-analysis. Primary and revisional RYGB had the same impact on DM and hypertension status after surgery. Based on our meta-analysis, we can assume that the metabolic effect is equal regardless of the previous bariatric treatment. However, this finding must be interpreted with caution because we were not able to provide any additional data on the severity of DM and hypertension. It is likely that there were differences between groups. Therefore, this issue needs to be investigated further.

In addition to primary outcomes, we decided to analyze the operative time and length of hospital stay. Unsurprisingly, revisional surgery takes longer (additional 44 min in the meta-analysis). In our opinion, this parameter is of little relevance since the surgeon’s experience was not analyzed in any way. It is obvious that revisions are considered more demanding, and for this reason, more advanced surgeons are selected. It is not clear whether it has any impact on clinical outcomes. There are studies showing that prolonged operative time may lead to the development of specific complications such as rhabdomyolysis [40].

The length of hospital stay was indifferent between groups. The *I*^2^ of 98% shows there are major differences in the postoperative stay in the hospital. None of the studies provided details on perioperative care. Taking into consideration the rapid changes in perioperative care and their influence on outcomes (introduction of enhanced recovery protocols), we consider this parameter to be no longer a reliable clinical benchmark [41, 42].

This review has some rather obvious shortcomings. It comprises only retrospective studies. However, this is the best available evidence since randomization is not possible. We did not analyze indications for revisional surgery (weight regain/complications of primary surgery), and perhaps, this aspect and better patient selection might help in improving the quality of future evidence. Moreover, we did not find information on the surgeon’s experience and institutional volume as well as the operative technique used. We assume that there might have been variation in the experience of surgeons performing primary and revisional surgery, since the latter is considered more difficult. This might have contributed to biased results. Four studies included patients undergoing both laparoscopic and open surgery which, to some extent, might have biased the results. We realize that the majority (> 95%) of RYGB are currently performed laparoscopically; therefore, further analyses should perhaps select only minimally invasive cases. However, when choosing inclusion criteria and building search strategy, surgical access was not limited and so we decided to follow initial assumptions. Additionally, our study did not analyze the initial BMI before primary surgeries, which may affect EWL after revisional procedures.

## Conclusion

This is the first systematic review attempting to show that revisional RYGB is associated with worse short- and long-term surgical outcomes when compared to primary procedures. It includes case-control and case-matched studies. The quality of included studies in general was moderate. Despite the higher morbidity and mortality in revisional group, these parameters are still relatively low. Moreover, the worse bariatric effect in terms of excess weight loss was observed after revision, but there were no differences in the resolution of obesity-related diseases. Therefore, it seems that revisional RYGB will still play a significant role as secondary procedure in patients who failed to lose weight or developed complications after primary surgery. In order to see which patients will particularly benefit from revisional RYGB, further studies focused on strict inclusion criteria and indications to surgery are required.

## Electronic Supplementary Material

ESM 1 (PNG 32 kb)

ESM 2 (GIF 17 kb)

High Resolution (EPS 91 kb)
