# Supplementary figures and images for: Revisional Gastric Bypass Is Inferior to Primary Gastric Bypass in Terms of Short- and Long-term Outcomes—Systematic Review and Meta-Analysis
Source: Obes Surg. 2018 May 11;28(7):2083–91. doi: 10.1007/s11695-018-3300-2 (PMC6018598; doi:10.1007/s11695-018-3300-2)

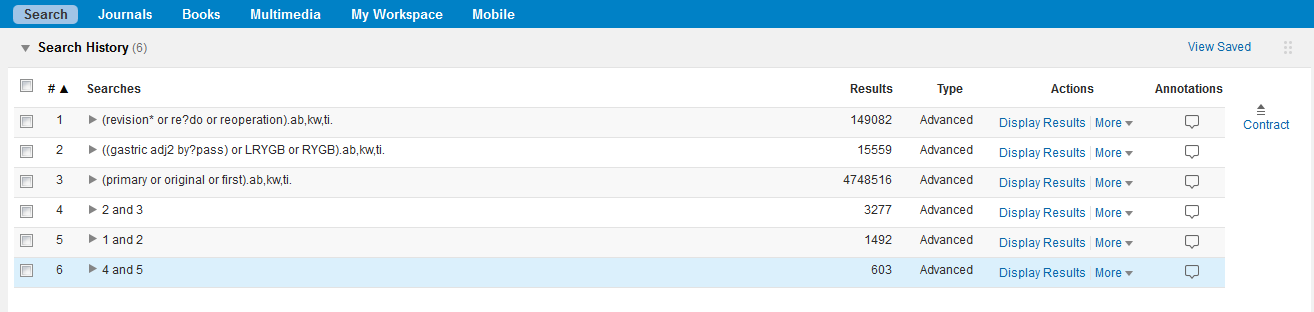

Supplement: Supplementary file 1 — (PNG 32 kb) [file 11695_2018_3300_MOESM1_ESM.png]

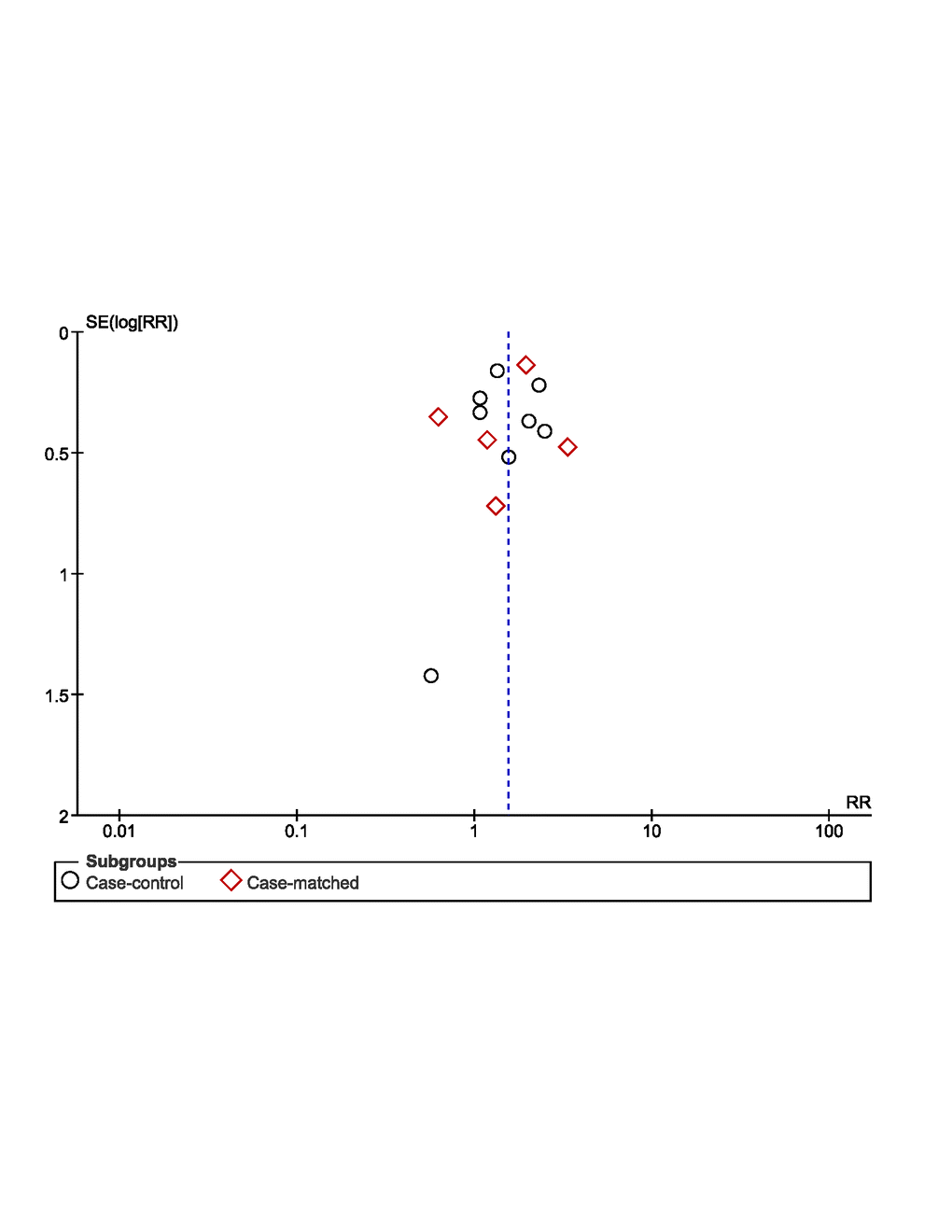

Supplement: Supplementary file 2 — (GIF 17 kb) [file 11695_2018_3300_Fig10_ESM.gif]
